# Occupational consequences after isolated reconstruction of the insufficient posterior cruciate ligament

**DOI:** 10.1186/1756-0500-7-201

**Published:** 2014-03-31

**Authors:** Christoph Ihle, Atesch Ateschrang, Dirk Albrecht, Johannes Mueller, Ulrich Stöckle, Steffen Schröter

**Affiliations:** 1 Department of Traumatology and Reconstructive Surgery, Eberhard Karls University Tübingen, BG Trauma Center Tübingen, Schnarrrenbergstr. 95, 72076 Tübingen, Germany; 2 Group practice, Traumatology and Reconstructive Surgery, Listplatz 1, 72764 Reutlingen, Germany; 3 Department of Gastroenterology, Klinikum Ludwigsburg, Posilipostraße 4, 71640 Ludwigsburg, Germany

**Keywords:** Posterior cruciate ligament, PCL reconstruction, Occupational consequence, Incapacity of work, Chronic PCL insufficiency, Health-related quality of life

## Abstract

**Background:**

With incorrect or even without treatment, acute injuries of the posterior cruciate ligament (PCL) can lead to chronic instability of the knee joint. After delayed treatment, negative occupational changes and reduced quality of life can occur. These aspects have not yet been investigated. The purpose of this study was to evaluate occupational consequences after isolated reconstruction in cases of chronic PCL insufficiency.

**Findings:**

12 patients treated with PCL reconstruction in a single bundle technique, using hamstring tendon grafts, were evaluated. All patients were operated upon at least 3 months after injury. Mean time of follow-up was 51 ± 18.2 months (14–75). Radiological assessment (Telos stress device) showed a side comparison of total translation of 4.5 ± 2.6 mm. Occupational consequences have been evaluated by the classification system “REFA”. Median time incapacity for work was 8 weeks. Nearly all patients achieved the mental status of the normal population (SF-36), but physical status was still restricted. A pre- to postoperative improvement of the clinical scores could be seen: Lysholm-Score: 46.4 ± 17.3 to 84.7 ± 14.1, HSS-Score: 74.3 ± 10.5 to 88.3 ± 10.7. Postoperative evaluated scores were: Tegner score: 4.8 ± 1.2, IKDC score: 80.0 ± 16.2, VPS: 3.4 ± 2.7. Patients with low physical load in their workplace described significantly better clinical results in every clinical score (p < .05) and less pain than patients with high physical load prior to the accident (VPS: REFA < 2: 2.4 ± 2.6, REFA ≥ 2: 5.5 ± 1.7; p < 0.05).

**Conclusions:**

Operative treated patients with a chronic PCL insufficiency achieve an improvement of the clinical result. Patients with low physical load at their workplace achieve less restrictions.

## Findings

### Introduction

In recent years, injuries of the posterior cruciate ligament (PCL) have received more attention. The incidence of PCL tears in trauma patients with acute haemarthrosis of the knee is stated to be between 8.1% and 38% [1,2]. Approximately half of patients with PCL tears have combined multiple ligament injuries (52%) [2]. As a result, many centres routinely perform reconstructions of the PCL. Based on the initial diagnosis and associated structures, different surgical techniques and different grafts are used [3-12]. With surgical treatment, good clinical results and improved subjective patient outcomes can be achieved [13-17]. In isolated PCL injuries, operative treatment is still a point of controversy [18,19], and a consistent survey of guidelines for the postoperative management of PCL injuries is still missing [20,21]. Although it is a frequent lesion, many injuries are not diagnosed and treated immediately [22,23]. Based on the period of time between the accident and surgical treatment, the classification into acute (under 3 months) and chronic (more than 3 months) is widespread [24]. With incorrect or even without treatment, acute injuries can lead to chronic instability and degenerative changes of the knee joint [25]. Deficiency of the PCL can lead to abnormal loading situations, especially of the patellofemoral joint kinematics, and can predispose the cartilage to degenerative changes [26]. Occupational consequences (long period of disability, need of occupational retraining, and low work intensity after reconstruction) and reduced quality of life can occur. This subjective quality of life as a new endpoint, also in knee surgical areas, has become an important factor in the rating of treatment results [27]. Therefore, the SF-36 (Short Form) Health Survey is an internationally accepted instrument. Mental health is considered at the same level as physical results. However, occupational consequences, incapacity of work and the quality of life after the reconstruction of chronic (treatment over 3 months) isolated PCL injuries have not been published so far.

The purpose of this study was to evaluate the clinical and radiological outcome after the surgical treatment of isolated PCL tears and chronic instability. Specific attention was given to occupational consequences and the quality of life of the patients. The hypothesis that operative treated patients with an isolated PCL injury and chronic instability achieve an improvement of the clinical result and norm values of quality of life with no occupational restrictions was established.

## Methods

This study was performed with the approval of the local ethics committee. A written consent of the study participants is present. All patients with the diagnosis “isolated rupture of the PCL” were included. All patients had to be treated in the same hospital (01/2003-12/2007) with an operative arthroscopic reconstruction of the PCL using hamstring tendon grafts. During this period, 90 patients with a PCL lesion were treated in the same hospital. All patients who declined to participate in this study, those who received operative treatment for less than 3 months or those who had already undergone a reconstruction of the PCL in the past were excluded. Patients who received their injury in the course of a polytrauma were also excluded, because of multiple comorbidities influencing the clinical result or the subjective health-related quality of life. In order to obtain a homogeneous group of patients, PCL avulsion fractures, medial or lateral collateral ligament ruptures or simultaneous anterior and posterior cruciate ligament injuries were also excluded. The minimum time of follow-up was 12 months after operative treatment. After exclusion, a homogeneous sample of 12 patients was left. The participants (17% female, 83% male) treated with a PCL reconstruction were evaluated by clinical and radiological examination (12/2008-01/2010); in 58%, the left side was affected. The most important causes of injury were sports activities (42%), followed by traffic accidents (25%) and occupational accidents (25%). Mean time of follow-up was 51 ± 18.2 months (14–75), mean age at the time of accident was 28.2 ± 9.9 years (15–43) and at time of follow-up examination was 34.8 ± 9.8 years (20–51). Mean body mass index was 25.8 ± 4.0, and 50% were smokers. For all study participants, the interval from accident to surgery was over 3 months.

### Surgical procedure

All patients were treated with a surgical reconstruction of the ruptured PCL; in every case, hamstring tendon grafts were used. Surgery was performed with the patients under spinal or general anaesthesia. Single-shot antibiotics and prophylactic low-dose heparin were used. Initially, diagnostic arthroscopy was performed to evaluate the meniscal, cartilage and ligamentous injuries. The surgical technique consisted of harvesting the hamstring tendon through an anteromedial incision. Usually, if the length of the semitendinosus tendon was 27 cm or longer, only one tendon was used; otherwise the gracilis tendon was prepared as well. A single-bundle technique with a tendon graft diameter of 7.5 mm to 9 mm was performed. The PCL origin was prepared by positioning a K-wire with a distance of 5 mm to the cartilage zone, over drilling the K-wire 3 mm less to the required diameter of the tendon graft, followed by dilatation to the size of the tendon graft. Through a posterior portal, the dorsal tibial rear edge and the insertion of the PCL was prepared, by positioning the guiding device in the centre of the PCL insertion, drilling a K-wire and over drilling to the size of the graft and dilatation to 0.5 mm more. Insertion of the tendon graft was performed with a shuttle suture, fixation with a femoral Endobutton (Aesculap AG, Tuttlingen, Germany) and a tibial interference screw Milagro (Mitek, DePuySynthes, Norderstedt, Germany) and an additional suture disc (Aesculap AG, Tuttlingen, Germany) with anterior tibial stress.

Postoperatively, the patients were allowed 20 kg partial weight bearing on the side of surgery using two crutches and a brace FIX AT4® (ORMED GmbH, Freiburg, Germany) in 10° extension with a posterior truss pad (in-house production). Passive exercise in prone position was allowed. After 2 weeks, the brace was changed to PCL brace 4TITUDE® (ORMED GmbH, Freiburg, Germany) with a limit of 90° flexion and active physiotherapy was started. During the night, the brace in 10° extension was required. After 6 weeks, the patient was allowed to full weight bear, and the PCL brace changed to no limit. After 3 months, no brace was used.

### Clinical assessment

The in-patient duration was obtained out of the patient charts. Hospital for Special Surgery (HSS) score [28,29], Lysholm-Gillquist score [30], IKDC Score, Visual pain scale (VPS) and Tegner activity score [31] were used for clinical assessment. The HSS score (62% subjective, 38% objective) includes 6 subscales with 7 items. Maximum value amounts to 100 points. The Lysholm-Gillquist knee functional scoring scale (95% subjective, 5% objective) and IKDC Score were assessed by the patients as a self-administered questionnaire. The Lysholm-Gillquist Score consists of eight items, in which pain and instability account for 30 of the total score of 100 points. The IKDC Score consists of three subscales, with the total score being 100 points. The subscales describe symptoms, sporting activity and function. The Tegner activity level covers activities of daily living, recreation, competitive sports and work.

The SF-36 health survey consists of 36 questions and represents the subjective health-related quality of life in the form of 8 scales. In this “health-related quality of life of the patient”, a high value is placed on the patients mental health and not only on his physical wellbeing. 4 Scales are used to describe the physical status (PF, RP, BP, GH), while 4 other scales (VT, SF, RE, MH) place their focus on describing the mental status of the patient. Transforming the results into norm-based scoring, each scale is comparable to the norm population and to other patient groups. Based on this norm-based scoring by Ware [32], a comparative interpretation including the gender and age structure is now possible. The score of the respective norm sample is 50. A norm-based scoring of the study participants over 50 is above average, while a score under 50 is below the average of the respective norm sample.

The German classification system “REFA” from occupational medicine and social medicine (Table 1) was used to classify the workload. The REFA-Classification is already used in knee surgery to describe changes in the workplace after operative intervention [33]. However, it is a useful instrument that should also be established in the cruciate ligament surgery. The work intensity before the accident and after reconstruction according the REFA classification was described.

**Table 1:** REFA classification

**Grade**	**Work intensity**	**Example**
**0**	Work without special physical strain	Work without load, like desk work
**1**	Work with small physical strain	Handling light work pieces; also lengthy standing or walking around
**2**	Work with moderate physical strain	Handling of 1–3 kg control device; carrying loads of 10–15 kg; climbing stairs or ladders without load
**3**	Work with hard physical strain	Carrying loads of 20–30 kg, shovelling, digging, chipping, climbing stairs or ladders with moderate load, moderate work in tense work posture
**4**	Work with most heavy physical strain	Carrying loads of more than 50 kg, climbing with heavy load, Hard work in tense work posture

**Figure 1:**
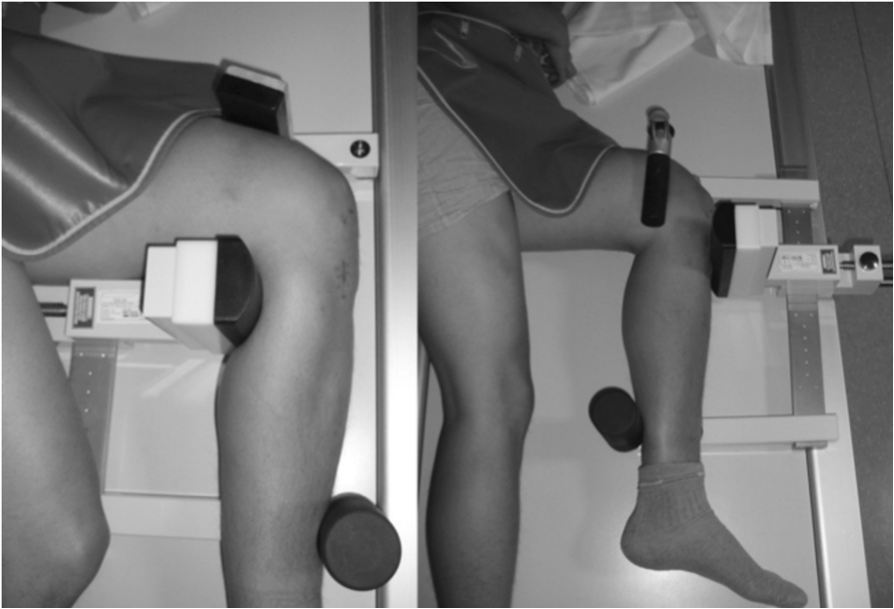
**The patient is positioned in the Telos stress device to the anterior stress (left) and the posterior stress (right) with an applied load of 20 kp, described by others **[34]**,**[35]**.**

**Figure 2:**
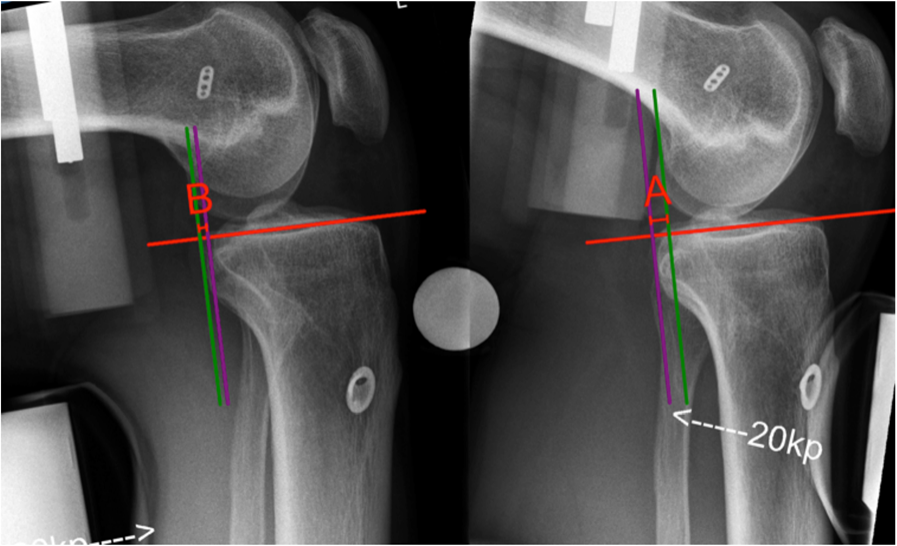
Posterior tibial displacement (A) and anterior tibial displacement (B) on stress radiograph with an applied load of 20 kp.

Stress radiographs in the lateral view of both knee joints were performed by the Telos stress device (METAX, Kupplungs- und Dichtungstechnik GmbH, Germany). To assess the radiological results (anterior, posterior and total translation), both knees were measured in 90° flexion and quantified in the scaled radiographs with mediCAD® (Hectec GmbH, Germany) (Figures 1 and 2). All scores and clinical examinations were assessed by one examiner (C.I.).

### Statistical analysis

All collected data were recorded in a Microsoft Access 2007 database. Statistical analysis was performed by JMP 10.0.0. Pre- to postoperative HSS- and Lysholm-Gillquist Score were compared with the paired Student *t* test. Radiographic results were compared in all patients with the paired Student *t* test. The clinical results between the patients with low and high physical load at the workplace were compared with ANOVA. The level of significance was α=0.05.

## Results

### Radiographic results

The results of the total translation (stress radiographs, posterior to anterior displacement) of the affected side, the opposite side and the side comparison in the stress radiographs (lateral view) are displayed in Table 2. The side comparison of the total translation was 4.5 ± 2.6 mm and showed a significant difference between the affected and healthy opposite side. Regarding the working load previous to the accident, there is no difference of total tibial translation after operative intervention.

**Table 2:** Radiographic results

	**n**	**Anterior tibial displacement**			**Posterior tibial displacement**			**Total tibial displacement**		
		**Injured side**	**Healthy side**	**Side difference**	**Injured side**	**Healthy side**	**Side difference**	**Injured side**	**Healthy side**	**Side difference**
Total	12	0.8 ± 2.7	0.6 ± 3.3	2.3 ± 2.6	−7.2 ± 3.4	−1.9 ± 1.9	5.2 ± 2.6*	7.8 ± 2.9	3.3 ± 2.3	4.5 ± 2.6*
REFA < 2	8	0.8 ± 2.9	0.0 ± 3.7	2.9 ± 2.9	−6.8 ± 3.0	−1.6 ± 1.9	5.1 ± 2.7	7.4 ± 2.7	2.8 ± 2.3	4.6 ± 2.9
REFA ≥ 2	4	0.8 ± 2.6	1.8 ± 2.5	1.0 ± 0.8	−8.0 ± 4.4	−2.5 ± 1.7	5.5 ± 2.9	8.8 ± 3.6	4.3 ± 2.1	4.5 ± 2.1

Tibial Displacement in the sagittal plane (anterior, posterior and total) in mm. *significant difference.

### Occupational consequences

The median time incapacity for work of all patients was 8 weeks. 17% of the patients could no longer exercise their occupation in the primary way, and occupational retraining was necessary. 83% could maintain the same physical workload as prior to the accident (Table 3). REFA prior to the accident in connection with the period of incapacity for work after reconstruction showed a significant difference between patients with low (REFA < 2) and high (REFA ≥ 2) physical workload (p < 0.05). The median period of incapacity for work for REFA < 2 was 6 weeks and 24 weeks for REFA ≥ 2. 17% of the patients lost one severity level (Table 3).

**Table 3:** Incapacity for work and REFA-Classification

	**Number**	**Incapacity for work**	**Occupational retraining**		**REFA shift of level of severity**		
		**Median (Range)**	**Yes**	**No**	**0**	**−1**	**−2**
Total	12	8 (2–52)	17%	83%	83%	17%	0%
REFA < 2	8	6 (2–24)	12.5%	87.5%	87.5%	12.5%	0%
REFA ≥ 2	4	24 (8–52)*	25%	75%	75%	25%	0%

*Significant difference between low and high working load (p < 0.05).

### Clinical results

The results of the health-related quality of life are displayed in Tables 4, 5 and 6. Considering all patients (Total), the mental status of the respective norm population could be achieved. Physical Functioning, Role Physical and Bodily Pain, three of the scales describing the physical status, still have differences to their respective norm samples; Bodily Pain shows a significant difference. Overall, the physical scales show a tendency to lower results than those describing mental status. For patients with high physical load in the workplace prior to the accident (REFA ≥ 2), pain is the most important factor reducing the physical part of the health-related quality of life. There is also a restriction in the other scales of physical status. The mental results were comparable.

**Table 4:** SF-36 physical health

		**Physical function**		**Role-physical**		**Bodily pain**		**General health**	
		**Mean ± SD**		**Mean ± SD**		**Mean ± SD**		**Mean ± SD**	
	**n**	**HP**	**NBS**	**HP**	**NBS**	**HP**	**NBS**	**HP**	**NBS**
Total	12	85.4 ± 19.0	44.1 ± 12.8	79.2 ± 38.2	44.9 ± 15.4	64.3 ± 26.3	41.1 ± 10.4	80.4 ± 16.0	54.0 ± 8.6
REFA < 2	8	91.9 ± 17.1*	49.7 ± 8.9*	90.6 ± 26.5	50.2 ± 8.6*	74.4 ± 23.6*	45.8 ± 8.6*	79.9 ± 19.5	53.6 ± 10.2
REFA ≥ 2	4	72.5 ± 17.6*	33.0 ± 12.8*	56.3 ± 51.4	34.4 ± 21.9*	44.3 ± 20.9*	31.7 ± 6.7*	81.5 ± 7.1	54.8 ± 5.5

HP: Health profile; NBS: Norm based scale.

*Significant difference of REFA < 2 to REFA ≥ 2 (p < .05).

**Table 5:** SF-36 mental health

		**Vitality**		**Social function**		**Role-emotion**		**Mental health**	
		**Mean ± SD**		**Mean ± SD**		**Mean ± SD**		**Mean ± SD**	
	**n**	**HP**	**NBS**	**HP**	**NBS**	**HP**	**NBS**	**HP**	**NBS**
Total	12	64.2 ± 17.6	48.5 ± 9.8	87.3 ± 21.5	47.5 ± 12.6	91.7 ± 28.9	48.8 ± 14.9	75.0 ± 21.5	49.3 ± 13.1
REFA < 2	8	68.8 ± 17.9	51.1 ± 10.1	93.6 ± 13.6	51.3 ± 7.7	87.5 ± 35.4	46.7 ± 18.2	77.5 ± 20.3	51.1 ± 12.1
REFA ≥ 2	4	55.0 ± 14.7	43.1 ± 7.6	74.8 ± 30.9	39.9 ± 18.1	100 ± 0.0	53.1 ± 0.5	70.0 ± 26.2	45.7 ± 16.1

HP: Health profile; NBS: Norm based scale.

**Table 6:** SF-36: differences between norm-based scale and norm-based population

	**Total**	**REFA < 2**	**REFA ≥ 2**
	**Mean ± SD (p-value)**	**Mean ± SD (p-value)**	**Mean ± SD (p-value)**
Physical function	−5.9 ± 12.8 (n.s.)	−0.3 ± 8.9 (n.s.)	−16.9 ± 12.9 (<0.05)
Role-physical	−5.1 ± 15.4 (n.s.)	0.2 ± 8.6 (n.s.)	−15.6 ± 21.9 (n.s.)
Bodily pain	−8.9 ± 10.4 (<0.05)	−4.1 ± 8.6 (n.s.)	−18.3 ± 6.7 (<0.05)
General health	4.0 ± 8.6 (n.s.)	3.6 ± 10.2 (n.s.)	4.8 ± 5.5 (n.s.)
Vitality	−1.5 ± 9.8 (n.s.)	1.1 ± 10.1 (n.s.)	−6.9 ± 7.6 (n.s.)
Social function	−2.5 ± 12.6 (n.s.)	1.3 ± 7.7 (n.s.)	−10.1 ± 18.1 (n.s.)
Role-emotion	−1.2 ± 14.9 (n.s.)	−3.3 ± 18.2 (n.s.)	3.1 ± 0.5 (n.s.)
Mental health	−0.7 ± 13.1 (n.s.)	1.1 ± 12.1 (n.s.)	−4.3 ± 16.1 (n.s.)

The results of the pre- and postoperative raised clinical scores are displayed in Table 7. A significant improvement of the pre- to postoperative value could be shown in Lysholm-Gillquist-Score and HSS-Score. Considering the results, good (Lysholm-Gillquist Score) to very good (HSS-Score) subjective and objective clinical results could be achieved. 92% of the patients could be grated into category “very good” or “good” (HSS-Score), while no patient remained in the “poor” category with a result under 60 points postoperatively.

**Table 7:** Clinical scores

	**Total (n = 12)**	**Total (n = 12)**	**REFA < 2 (n = 8)**	**REFA ≥ 2 (n = 4)**
	**Mean ± SD**	**Median (range)**	**Mean ± SD**	**Mean ± SD**
IKDC preop	---	---	---	---
IKDC postop	80.0 ± 16.2	85.0 (47.1-97.7)	86.9 ± 12.3	66.1 ± 15.1°
VPS preop	---	---	---	---
VPS postop	3.4 ± 2.7	3.0 (0.0-7.0)	2.4 ± 2.6	5.5 ± 1.7°
LG Score preop	46.4 ± 17.3	50.5 (14.0-73.0)	50.0 ± 19.0	40.0 ± 12.0
LG Score postop	84.7 ± 14.1*	90.0 (61.0-100.0)	89.5 ± 12.3	75.0 ± 13.4°
Tegner preop	---	---	---	---
Tegner postop	4.8 ± 1.2	5.0 (3.0-8.0)	5.1 ± 1.2	4.0 ± 0.8
HSS Score preop	74.3 ± 10.5	78.0 (51.0-86.0)	81.4 ± 3.2	65.5 ± 9.7°
HSS Score postop	88.3 ± 10.7*	90.5 (64.0-100.0)	93.8 ± 5.9	77.3 ± 9.9°

*Significant improvement of the pre- to postoperative value (p < 0.05).

°Significant difference of REFA < 2 to REFA ≥ 2 (p < 0.05).

Nevertheless, the examined patients were still not free of pain. 50% reported having knee pain at least occasionally; 17% were dependent on the regular use of analgesics. Patients with low physical load at their workplace described, beside better clinical results, significantly less pain than patients with high physical load previous to the accident (Table 7).

## Discussion

The current work described the incapacity for work (8 weeks) and the relation to the workload according to the REFA classification (6 weeks REFA < 2, 24 weeks REFA ≥ 2) after the surgical treatment of chronic PCL insufficiency of the 12 included patients. The patients nearly achieved the quality of life mental status of the normal population (SF-36), but even the physical status was still more limited than the mental field. For patients with high physical load at the workplace, pain is the most important factor reducing the physical field of the subjective quality of life. With the applied surgical technique, good clinical results can be achieved. For patients with high physical load at their workplace, pain is still a problem and more occupational restrictions have to be expected after surgery. Based on the presented data, the study was able to show the importance of the physical load at the workplace prior to the accident. To get better postoperative results, the operative treatment of patients with high muscle load and a great range of motion in their daily activity should be adapted. Kinematics in all 6 degrees of freedom and under physiological muscle load situation should be considered in the planning of operative treatment.

The REFA-Classification was selected to record occupational consequences of the study participants. The authors are not aware of anyone having applied the REFA-Classification in cruciate ligament surgery in the past. It is an established instrument in other fields of knee surgery (open wedge HTO) to record sensitive changes of the physical load at the workplace, especially when there is no need for retraining [33]. The study results revealed a tendency to workplaces with a lower physical load (17%). The median period of incapacity for work was 8 weeks. The results of the study showed the relevance of the preoperative working load of the patients for the postoperative occupational consequences. Patients with low physical load at their workplace prior to the accident had less postoperative occupational restrictions. For most of these patients (REFA < 2), occupational retraining is not necessary (87.5%) and significant previous re-entry to occupational life is possible. Hirschmann et al. [36] evaluated the outcome after PCL reconstruction of bicruciate ligament injuries. 48 out of 68 patients received a surgery within 2 weeks, nearly all participants were treated acutely (< 3 months). Similar results could be shown for the need for retraining (82% of the patients returned to their previous work). A differentiation of the physical load in the workplace prior to the accident and after treatment was not analysed. To include these sensitive changes, the REFA-Classification was used in the present study. Hirschmann et al. [36] reported a mean time to return to work of 9 ± 13 months. These less positive results can be explained by the more severely injured patients with bicruciate ligament lesions. In summary, patients with low physical load at their workplace (REFA < 2) can re-enter occupational life earlier and have less occupational restrictions than patients with a high work intensity (REFA > 2) prior to the accident. Whether early treatment (<3 months) can prevent negative occupational consequences is still not known and there is a need for further studies.

So far, the subjective health-related quality of life has found no essential attention in the posterior cruciate surgery. The present study showed that nearly standard values of the mental health of quality of life could be achieved by reconstructing isolated PCL injuries. In physical health, restrictions are still expected. Pain is the most limiting factor of the physical status, especially for patients with high physical load at their workplace previous to the accident. A connection between the working load prior to the accident and the physical part of the health-related quality of life could be shown. Patients with lower physical load (REFA < 2) at the workplace have significantly less restrictions in their postoperative physical health than patients with REFA≥2. By analysing the clinical long-term outcome of surgically treated knee dislocations (bicruciate ligament injuries), Hirschmann et al. [36] showed a mean SF-36 result of 81 ± 15 (Mental health: 54 ± 8, Physical health 50 ± 7). Most of the patients were treated acutely (<3 months). It is not clear whether the reported data is norm-based or not. The lower result for physical health in comparison to mental health is comparable to our data. Schofer et al. [37] also reported about reduced physical health after simultaneous bicruciate reconstruction. Most of the patients were treated chronically (average time from injury to surgery: 235 days). 9 patients were evaluated retrospectively using the SF-36 questionnaire, with an average time of follow-up of 37 months. Lower values were shown. These less positive results can be explained by the more severe bicruciate lesions, also. Conversion into norm-based scoring was not performed. Thus, direct comparison is not possible because of different age distributions. Sekiya et al. [38] reported SF-36 Health survey results after the reconstruction of isolated chronic grade III PCL injuries. Based on a 6 year follow-up, they showed a total SF-36 result of 98 points with minimal limitations in their quality of life. The demonstration based on 1 single SF-36 value is contrary to common international practice and prohibits meaningful comparisons. Also, the demonstration with two total scores (mental health and physical health) was not used in this study because of methodical reservations [39]. It is not known whether early (<3 months) treatment has any influence on the postoperative health-related quality of life.

To determine the postoperative stability of the knee joint, Telos stress device was used as an established method [34]. The radiological stability testing showed comparable results of the group with high working load comparing to patients with lower working load prior to the accident. The side comparison of the total translation of all patients was 4.5 ± 2.6 mm. Despite good clinical results, a significant difference in the stability of the healthy knee joint of the opposite side could be shown. The stability of the healthy knee joint could not be reached, even though it is still a grade 0 PCL injury in the classification of Schulz et al. [40]. Ahn et al. [41] evaluated the stability after single-bundle transtibial PCL reconstruction using a bioabsorbable cross-pin tibial back side fixation using a Telos stress device. The follow-up period was comparable, with an average of 47 months. They showed a mean side to side difference in posterior translation of 3.2 ± 1.5 mm. Compared to the systematic review of the literature of Kim et al. [42], mean posterior knee laxity varied from 1.96 mm to 5.90 mm. Lahner et al. [14] described 3.2 ± 1.5 mm posterior knee laxity 4 years after isolated single-bundle PCL reconstruction. Yoon et al. [20] reported postoperative mean side to side differences of 2.5 ± 1.9 mm and 4.8 ± 2.4 mm after the reconstruction of isolated posterior cruciate ligament injuries comparing two methods for postoperative treatment (cast vs. brace). One possible explanation for the slightly inferior results is that that the present sample consists of only chronic-treated (>3 months) patients. It consists of many occupational accidents, mostly overweight patients (Mean body mass index over 25) and 50% smokers.

Although the patients are still not free of pain, the clinical scores show good to very good (Lysholm-Gillquist Score, HSS Score, IKDC Score) clinical results after the reconstruction of isolated PCL injuries. A significant improvement of the pre- to postoperative evaluated values (Lysholm-Gillquist Score, HSS Score) could be shown. Especially for patients with high physical load at their workplace (REFA≥2), pain is still a problem. Patients with low physical load at their workplace achieve better clinical results and describe a less painful situation. Lin et al. [43] described the postoperative pain situation after using patellar tendon vs. hamstring tendon grafts. However, whether the patients were treated early (<3 months) or chronically is not known. For patellar tendon autografts, a higher frequency of knee pain could be shown. Lahner et al. [14] reported comparable clinical data after the reconstruction of isolated PCL injuries using the arthroscopic single bundle technique. The mean time of follow-up was 47.2 ± 8.7 months. They showed a postoperative Tegner activity score of 5.7 ± 1.3 points and an IKDC of 65.1 ± 13.5 points. There was no differentiation between acute and chronic treatment. The presented study confirms their results with a median Tegner activity score of 5 and a median IKDC score of 86.2. Min et al. [17] also evaluated the clinical results after double-bundle PCL reconstruction using a single-sling method with a tibialis anterior allograft. The medium follow-up period was 49.2 months. A Lysholm-Gillquist Score of 83.5 ± 13 could be achieved. The pattern of injury of the evaluated patients is not clearly stated and a new, unestablished surgical technique was presented. This could be the reason for the lower results compared to the presented data. In a systematic review, Kim et al. [42] summarised the available literature of reconstruction of the PCL using an arthroscopically-assisted single-bundle transtibial technique in patients with isolated PCL tears. They stated the range of mean values of the Lysholm Knee Score between 81 to 100 points and Tegner activity score between 4.7 to 6.3 points. Besides the heterogeneous period of follow-up and study designs, the results are comparable to our study. They noted that despite surgical reconstruction, degenerative osteoarthritis was frequently found at the time of follow-up. The results of Gill et al. [44] strengthen these findings. They could show, by analysing the *in vivo* tibifemoral and patellofemoral kinematics in PCL reconstructed knees, that PCL reconstruction could achieve a normal posterior tibial translation in all flexion angles, but no normal mediolateral translation of the tibia and no normal patellar rotation and tilt. These kinematic findings could be a possible explanation for the development of cartilage degeneration after the reconstruction of an isolated PCL lesion. Degenerative osteoarthritis was not integrated in the presented study design, but is a possible explanation for the pain status after chronic treatment or after high physical load at the workplace and should be precisely analysed in further prospective long-term studies. Gill et al. [45] simulated the influence of muscle loads to the kinematic situation of knee joint stability after the reconstruction of isolated PCL injuries. They demonstrated that reconstruction did not restore normal translation at high flexion angles over 60 to 90°. These results are a possible explanation of the fact that the pain situation of patients with high working load in the workplace is greater than the pain situation of patients with lower working loads, although no differences in translation could be found in our data (Meassurement in 90° Flexion). Patients with high working load at the workplace have higher muscle loads and work in flexion angles over 90°. To get better postoperative results, the operative treatment of patients with high muscle loads and great range of motion in their daily activity should be adapted. Kinematics in all 6 degrees of freedom and under physiological muscle loads should be considered. To strengthen these findings, further clinical and biomechanical studies are needed.

### Limitations

The current study has several limitations. There are only 12 patients included in this study. The lack of a control group with conservative or acute treated patients and the absence of preoperative stability data are also limitations of the present study. The preoperative analysis of the cartilage situation is also missing. A prospective study design with more included patients, only one surgeon and predefined follow-up periods would strengthen the results. Acute treatment under 3 months should be evaluated and considered in future study designs. To compensate for these weaknesses, a very high value was placed on the homogeneity of the included patients. All of the patients had the same pattern of injury, were treated with the same surgical technique and underwent the same postoperative management. Despite the limitations, the present study describes important outcome aspects after the reconstruction of PCL injuries. It complements the physical outcome with occupational and mental aspects, compared to biomechanical studies. The fact that pain is still a problem after chronic operative treatment and the importance of the physical load in the workplace prior to the accident confirm the clinical relevance of this study.

## Conclusions

The results demonstrate that patients with low workload at their workplace achieve fewer negative occupational consequences and better clinical results after the isolated reconstruction of PCL injuries. To get better postoperative results, the operative treatment of patients with high muscle load and great range of motion in their daily activity should be adapted. Kinematics in all 6 degrees of freedom and under physiological muscle load situations should be considered in the planning of operative treatment. Therefore, further clinical and biomechanical studies are needed. Standard values of the mental health of quality of life could be achieved. In the medium-term, restrictions in the physical health of the subjective quality of life are expected. To give a recommendation for the time between injury and surgical reconstruction (in the first three months), prospective studies or at least retrospective studies with more patients are required.

### Ethical approval

Approval of the ethical review committee of the Eberhard Karls University Tübingen 394/2008BO2.

## Competing interests

The authors declare that they have no competing interests.

## Authors’ contributions

All authors contributed to the steps of processing patient history as well as writing and editing the manuscript in a significant way. SS and CI conceived the idea for the study/publication, planned the whole study and engaged in writing the manuscript. JM and DA provided expertise in collection of the data, statistics and graphical work. US, SS and AA edited and reviewed the manuscript. US gave advice throughout the project and reviewed the manuscript. Furthermore, US was involved in the planning and review process. All authors read and approved the final manuscript.
